# Clinical characteristics of myelin oligodendrocyte glycoprotein antibody-associated disease according to their epitopes

**DOI:** 10.3389/fneur.2023.1200961

**Published:** 2023-06-26

**Authors:** Jin Myoung Seok, Mi Young Jeon, Yeon Hak Chung, Hyunjin Ju, Hye Lim Lee, Soonwook Kwon, Ju-Hong Min, Eun-Suk Kang, Byoung Joon Kim

**Affiliations:** ^1^Department of Neurology, Soonchunhyang University Hospital Cheonan, Soonchunhyang University College of Medicine, Cheonan, Republic of Korea; ^2^Department of Neurology, Neuroscience Center, Samsung Medical Center, Seoul, Republic of Korea; ^3^Department of Neurology, Samsung Medical Center, Sungkyunkwan University School of Medicine, Seoul, Republic of Korea; ^4^Department of Neurology, Korea University Guro Hospital, Korea University College of Medicine, Seoul, Republic of Korea; ^5^Department of Neurology, Inha University Hospital, Incheon, Republic of Korea; ^6^Department of Health Sciences and Technology, Samsung Advanced Institute for Health Sciences & Technology (SAIHST), Sungkyunkwan University, Seoul, Republic of Korea; ^7^Department of Laboratory Medicine and Genetics, Samsung Medical Center, Sungkyunkwan University School of Medicine, Seoul, Republic of Korea

**Keywords:** central nervous system demyelinating diseases, myelin oligodendrocyte glycoprotein, autoantibodies, epitopes, immunoassay

## Abstract

**Background:**

The detection of myelin oligodendrocyte glycoprotein autoantibodies (MOG-Ab) is essential for the diagnosis of MOG-Ab-associated disease (MOGAD). The clinical implications of different epitopes recognized by MOG-Ab are largely unknown. In this study, we established an in-house cell-based immunoassay for detecting MOG-Ab epitopes and examined the clinical characteristics of patients with MOG-Ab according to their epitopes.

**Methods:**

We conducted a retrospective review of patients with MOG-Ab-associated disease (MOGAD) in our single center registry, and collected serum samples from enrolled patients. Human MOG variants were generated to detect epitopes recognized by MOG-Ab. The differences in clinical characteristics according to the presence of reactivity to MOG Proline42 (P42) were evaluated.

**Results:**

Fifty five patients with MOGAD were enrolled. Optic neuritis was the most common presenting syndrome. The P42 position of MOG was a major epitope of MOG-Ab. The patients with a monophasic clinical course and childhood-onset patients were only observed in the group that showed reactivity to the P42 epitope.

**Conclusion:**

We developed an in-house cell-based immunoassay to analyze the epitopes of MOG-Ab. The P42 position of MOG is the primary target of MOG-Ab in Korean patients with MOGAD. Further studies are needed to determine the predictive value of MOG-Ab and its epitopes.

## 1. Introduction

Myelin oligodendrocyte glycoprotein autoantibodies (MOG-Ab) have been identified in patients with various central nervous system (CNS) demyelinating diseases, including single or recurrent optic neuritis (ON), transverse myelitis (TM), acute disseminated encephalomyelitis (ADEM), encephalitis, and brainstem encephalitis (1, 2). MOG-Ab-associated disease (MOGAD) is considered a distinct condition from multiple sclerosis (MS) or neuromyelitis optica spectrum disorder (NMOSD), yet the factors contributing to its clinical diversity remain unclear. The age at onset of MOGAD was known to be a relevant clinical factor that children can have ADEM-like brain lesions more frequently, whereas adult patients tend to present with optic neuritis (3). Further, MOG-Ab positivity has been linked to the clinical course of MOGAD; children with a monophasic clinical course become MOG-Ab negative earlier than those with relapsing courses (4).

The detection of MOG-Ab is commonly performed using a live cell-based immunoassay with full-length human MOG by indirect immunofluorescence or flow cytometry methods (5). Given that these methods allow for the expression of MOG in its native conformation, the conformational epitopes of MOG could be important for the detection of specific human MOG-Ab (1, 6). The epitope most frequently recognized by MOG-Ab is known to be Proline42 (P42) within the CC’-loop of the extracellular MOG domain (6), however, the clinical implications of different epitopes recognized by MOG-Ab are largely unknown.

In this study, we aimed to establish an in-house cell-based immunoassay for the detection of different epitopes of MOG-Ab and evaluate the clinical characteristics of patients with MOGAD in relation to epitope reactivity.

## 2. Method

### 2.1. Patients and samples

We conducted a retrospective review of patients registered in the CNS inflammatory disease registry at our tertiary medical center, covering the period from 2018 to 2021. All enrolled patients were diagnosed with MOGAD based on a recent recommendation and tested positive for MOG-Ab (5); the detection of MOG-Ab in enrolled patients was conducted through an in-house cell-based assay of MOG-IgG (Supplementary methods). We collected baseline and clinical characteristics of patients with MOGAD, which included age at onset, sex, disease duration, type of clinical course, total number of relapses during the follow-up, the clinical presentation at onset, and the preventive treatment profile; considering their clinical symptoms and magnetic resonance imaging (MRI) lesions together, clinical presentation at onset of patients were assigned into five clinical presentations: ON, TM, brainstem encephalitis, cerebral syndrome or ADEM, and poly-regional (3). We also included samples from patients who provided their informed consent, which were stored in 300-ul aliquots in microfuge tubes at −80°C for later analysis.

The study was approved by the institutional review board of the Samsung Medical Center, and all participants provided written informed consent.

### 2.2. Generation of human MOG variants and molecular cloning

To determine the epitopes recognized by MOG-Ab, the responses to human MOG (hMOG) and hMOG variants were tested. The sites of variants in hMOG were selected, as described by Mayer et al. (6); the hMOG α1 variants R9G/H10Y, N31Q (N-glycosylation site), P42S (immunodominant human MOG epitope), R86Q (mouse/rat specific), and H103A/S104E (binding site of monoclonal antibody 8-18C5 and human MOG epitope) (6–8) were synthesized and cloned into the pIRES2 DsRed-Express2 vector (Takara, Shiga, Japan) by a commercial gene synthesis service (Bioneer, Daejeon, Korea).

### 2.3. Immunoassays for the detection of MOG-Ab binding to different MOG epitopes

The cell-based assay (CBA)-flow cytometry analysis was performed using Human embryonic kidney 293 (HEK293) cells transfected with each expression vector encoding the different hMOG variants described above. The expression of all hMOG variants proteins was confirmed by reverse transcription-polymerase chain reaction (RT-PCR) and western blotting (Supplementary Figure S1). Transfected cells were incubated with each patient’s serum at a dilution of 1:20 for 1 h at room temperature and then developed with Alexa Flour 488 conjugated anti-human IgG1 (Thermo Fisher, Waltham, MA, United States) for imaging with a fluorescence microscope (Eclipse 80i, Nikon, Tokyo, Japan) or mouse anti-human IgG1 (Thermo Fisher, Waltham, MA, United States) with Alexa Fluor 647 conjugated anti-mouse antibody (Thermo Fisher, Waltham, MA, United States) at 1:500 for flow cytometry (Verse, BD, Franklin Lakes, NJ, United States). Flow cytometry data were analyzed using the FACS Suite (BD, Franklin Lakes, NJ, United States). The binding percentages of each hMOG variants was calculated based on the ratio of median fluorescence intensity (MFI) between hMOG and each hMOG variants (% binding = ΔMFI of hMOG variants / ΔMFI of hMOG × 100, ΔMFI = MFI positive − negative cells). MOG-Abs from enrolled patients were classified into two groups according to the immunoreactivity to the P42 position (P42 group vs. non-P42 group).

The detailed cloning strategies and the methods of transfection, western blot, and FACS analysis are described in Supplementary methods.

### 2.4. Statistical analysis

The clinical characteristics of the enrolled patients were presented with appropriate summary statistics. Continuous data were shown as means with standard deviations or as medians with interquartile ranges (IQRs). Categorical variables were presented as absolute and relative frequencies. We analyzed the characteristics of patients with MOGAD according to the presence of immunoreactivity to Proline42. The chi-square test or Fisher’s exact test was used for categorical variables, whereas continuous variables were analyzed using Student’s *t*-test, the Mann–Whitney U test, or the Kruskal-Wallis test. All statistical analyses and graph plotting were performed using R software version 4.2.1 (ggplot2 package; R Foundation for Statistical Computing, Vienna, Austria). Statistical significance was defined as a two-tailed value of *p* <0.05.

## 3. Results

### 3.1. Clinical characteristics of MOGAD

A total of 55 patients (29 females, 52.7%) with MOGAD were included. The mean age ± standard deviation at onset was 39.7 ± 17.2 years. All patients were seropositive for MOG-Ab, and seronegative for anti-aquaporin-4 antibody. The median disease duration was 1.7 (IQR, 0.7–3.9). The median total number of relapses was 2.0 (IQR, 1.0–3.5). 47 of 55 patients had at least one year or more of follow-up, and ten of them (10/47 patients, 21.3%) showed a monophasic clinical course. ON was the most frequent clinical presentation at onset, followed by brainstem encephalitis, TM, and cerebral or ADEM. In MOGAD patients with monophasic clinical course, ON (7/10 patients) was also the most frequent clinical presentation at onset. Only one patient showed the poly-regional onset of MOGAD. Azathioprine and mycophenolate were commonly used immunosuppressants to prevent relapses in patients with MOGAD. The clinical characteristics of the enrolled patients are presented in Table 1.

**Table 1:** Demographic and clinical characteristics of patients with MOG-IgG according to the presence of immunoreactivity to Proline42.

	Total patients (*n* = 55)	Patients with MOG-IgG binding to Proline42 site (P42 group) (*n* = 12)	Patients with MOG-IgG binding to other sites (non-P42 group) (*n* = 12)	*p*-value^*^
Age at onset, year (SD)	39.7 (17.2)	31.6 (21.5)	39.1 (15.4)	0.260
Childhood-onset (<18-year-old) (%)	4 (7.3)	4 (33.3)	0 (0)	0.093
Adult-onset (%)	51 (92.7)	8 (66.7)	12 (100)	
Female (%)	29 (52.7)	9 (75.0)	5 (41.7)	0.214
Disease duration, year (IQR)	1.7 (0.7–3.9)	0.9 (0.3–3.3)	1.5 (0.2–3.9)	0.840
Monophasic clinical course^†^ (%)	10/47 (21.3)	2/11 (18.3)	0/10 (0)	0.476
The number of attacks (IQR)	2.0 (1.0–3.5)	2.0 (1.8–3.5)	2.5 (2.0–5.0)	0.722
Clinical presentation at onset (%)				0.676
ON	38 (69.1)	8 (66.7)	8 (66.7)	
TM	5 (9.1)	2 (16.7)	0 (0)	
BS encephalitis	7 (12.7)	1 (8.3)	2 (16.7)	
Cerebral syndrome or ADEM	4 (7.3)	1 (8.3)	2 (16.7)	
Poly-regional onset	1 (1.8)	0 (0)	0 (0)	
Preventive treatment (%)				0.605
Azathioprine	16 (29.1)	2 (16.7)	2 (16.7)	
Mycophenolate Mofetil	14 (25.5)	5 (41.7)	4 (33.3)	
Oral Prednisolone	3 (5.4)	0 (0)	2 (16.7)	
Rituximab	1 (1.8)	0 (0)	0 (0)	
Other^‡^	5 (9.1)	1 (8.3)	2 (16.7)	
None	16 (29.1)	4 (33.3)	2 (16.7)	

^†^The patients with 1 year or more follow-up periods were included only. ^‡^Cyclophosphamide (*n* = 1), tacrolimus (*n* = 1 in non-P42 group), methotrexate (*n* = 1), interferon-beta (*n* = 1 in P42 group), dimethyl fumarate (*n* = 1 in non-P42 group). ^*^P42 group vs. non-P42 group. MOG-IgG, anti-myelin oligodendrocyte glycoprotein immunoglobulin G antibody; SD, standard deviation; IQR, inter-quartile range; ON, optic neuritis; TM, transverse myelitis; BS, brainstem; ADEM, acute disseminated encephalomyelitis.

### 3.2. Clinical characteristics according to the P42 position binding

**Figure 1:**
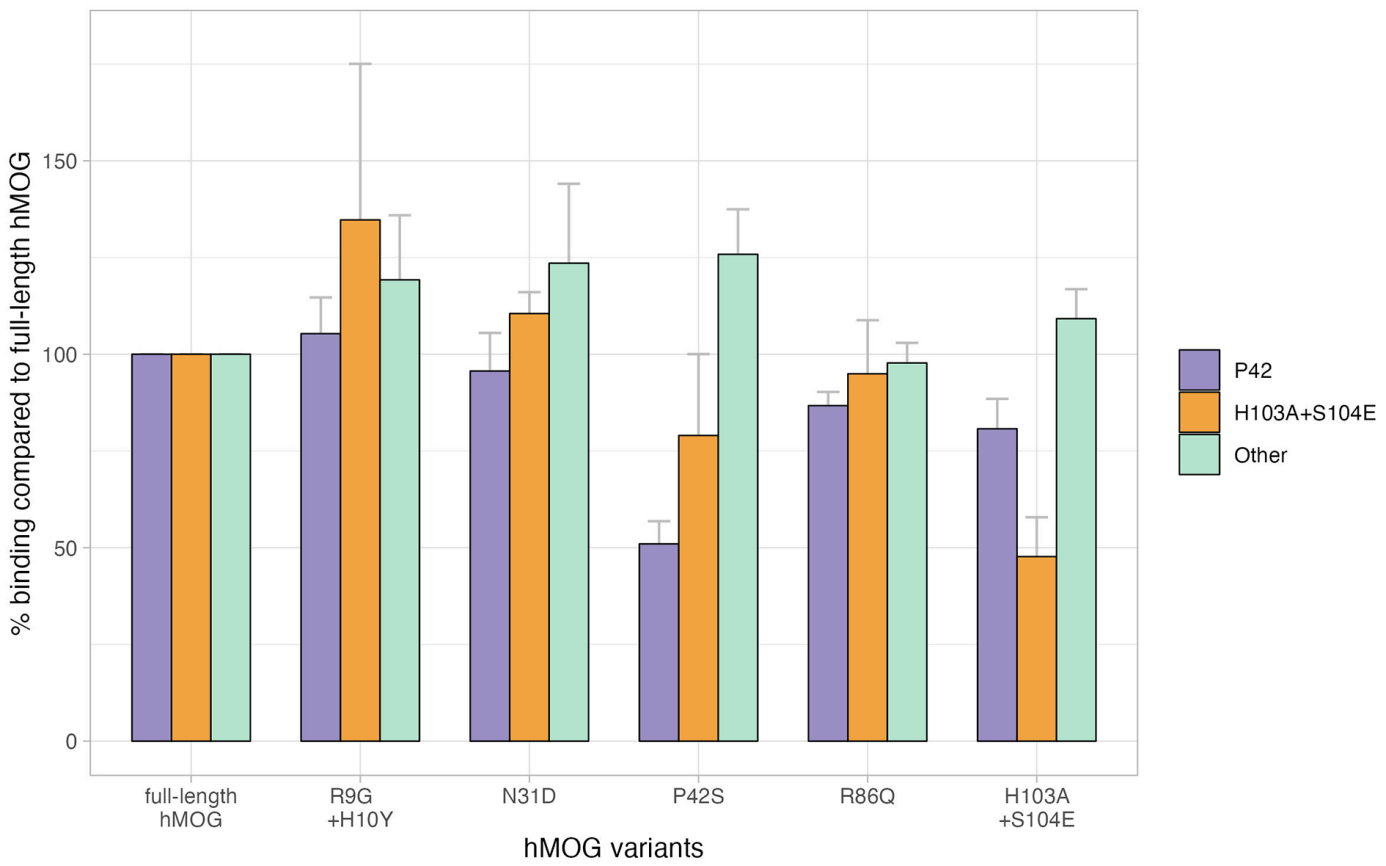
Different binding affinities to the position of MOG, Bar plots represent the percentage binding value of each human MOG variant compared to the human MOG; error bars represent standard error of the mean. There were 12 patients with MOGAD who showed reduced relative binding reactivity to P42S, suggesting that MOG-Ab recognizes the P42 position in MOG protein, and four patients with MOGAD had their MOG-Ab recognizing H103A + S104E. However, no specific epitopes were recognized in 10 patients with MOGAD showing preserved relative binding reactivity to human MOG variants.

**Figure 2:**
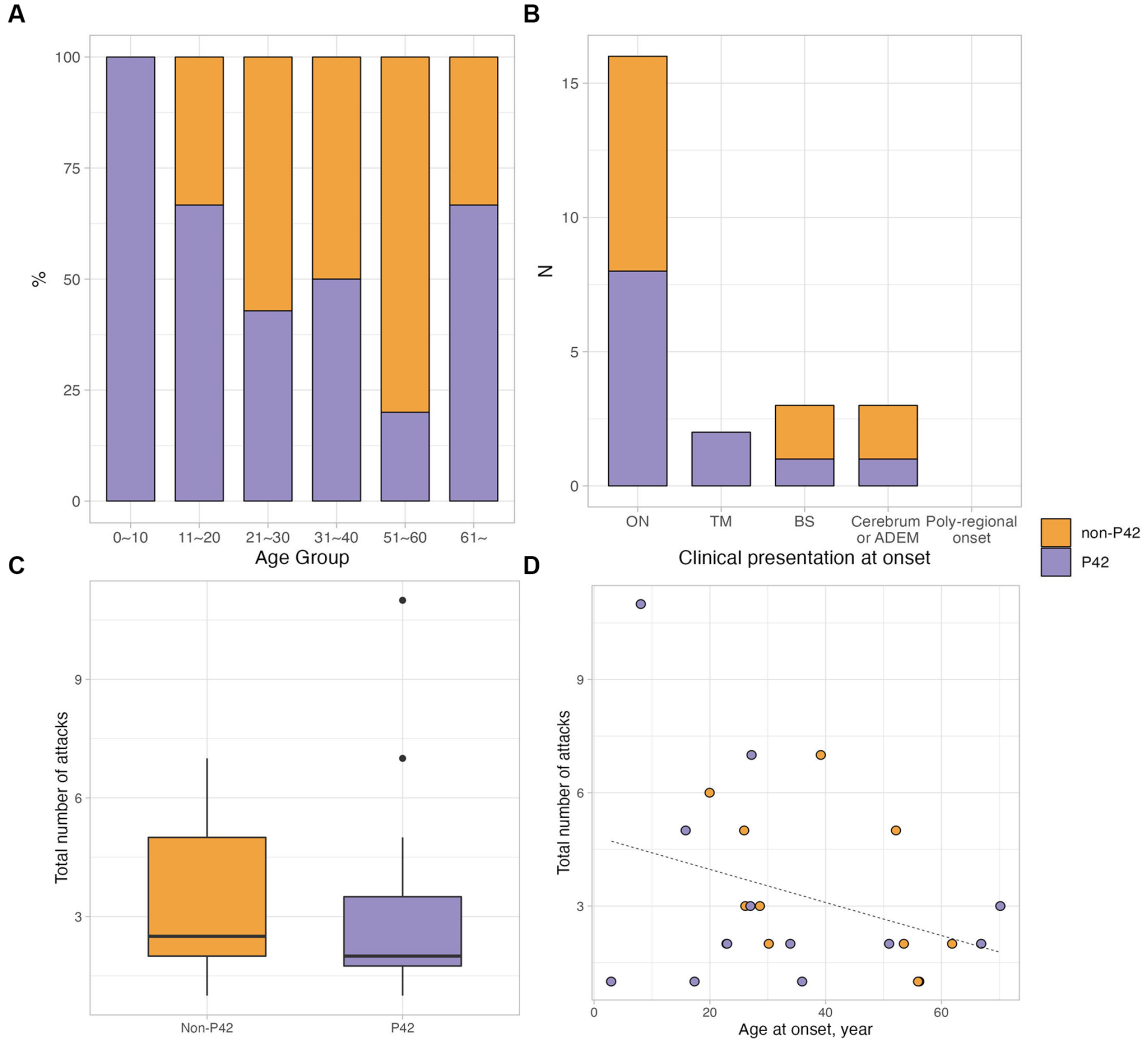
Clinical characteristics of patients with MOGAD according to the immunoactivity to P42. **(A,B)** The different proportions of P42 reactivity according to the age group and clinical presentation at onset. **(C)** The total number of attacks was lower in MOGAD patients having P42 reactivity, which was not significant (median 2.0, IQR 1.8–3.5 vs. median 2.5, IQR 2.0–5.0; *p* = 0.722). **(D)** The scatter plot showed the correlation between the total number of attacks and age at onset; in non-P42 group, there was a tendency of negative correlation between the total number of attacks and age at onset (rho = −0.560, *p* = 0.059).

Twenty four samples obtained from the 55 enrolled patients with MOGAD (female, 14/24, 58.3%; age of onset, 35.2 ± 18.8 years) were used for MOG-Ab epitope analysis. Out of 24 serum samples, 58.3% (14/24) exhibited a decreased binding reactivity to one or more of the human MOG variants. 10 samples demonstrated a reduction in binding to P42S, two samples to H103A/S104E, and the remaining two samples showed a decreased binding to both P42S and H103A/S104E human MOG variants. However, in ten samples from patients with MOGAD, there was no definite reduction in binding to human MOG variants (Figure 1). We divided the patients into two groups based on the presence of immunoreactivity to the P42 position of MOG protein: the P42 group and the non-P42 group. As shown in Table 1, 50% of samples from MOG-Ab positive patients were found to recognize Proline42 of MOG protein. The age of onset tended to be younger in the P42 group compared to the non-P42 group although it was not significant (31.6 ± 21.5 years vs. 39.1 ± 15.4 years, *p* = 0.260) (Figure 2). All four patients with childhood-onset MOGAD were found to have immunoreactivity to the P42 epitope. Additionally, 18.3% of patients in the P42 group had a monophasic clinical course, while none of the patients in the non-P42 group did. During follow-up, the P42 group had a lower total number of attacks compared to the non-P42 group. Both groups had optic neuritis as the most common clinical presentation at onset, and there were no significant differences of clinical presentations at onset between the groups. There were also no significant differences in sex or clinical phenotype during follow-up between the two groups based on the presence of P42 binding reactivity.

## 4. Discussion

In this study, we developed an in-house cell-based immunoassay to identify distinct epitopes of MOG-Ab. We also analyzed the clinical characteristics of patients with MOG-Ab who had varying binding reactivity to the epitopes of the MOG protein. This is the first study to investigate the epitopes of MOG-Ab in Korean patients with MOG-associated disorder (MOGAD) and examine the relationship between MOG-Ab epitopes and the clinical characteristics of MOGAD.

The clinical significance of MOG-Ab has been studied thoroughly with the discovery of reliable cell-based immunoassay for MOG-Ab using full-length human MOG protein and IgG1 antibody (9). The detection of MOG-Ab became an essential part of the differential diagnosis of CNS demyelinating diseases (5, 10), and it has helped to expand our understanding of the clinical diversity of MOGAD; the clinical syndromes of MOGAD include single/recurrent or bilateral ON, TM, ADEM, brain or brainstem syndrome, and cortical encephalitis (2, 5, 11–13). In our study, patients with MOGAD also showed diverse clinical syndromes, and ON was the most common presenting clinical syndrome (38/55, 69.1%), which was in line with the results of previous studies (3, 14, 15). Double seropositive cases, where patients have both aquaporin-4 autoantibody and MOG-Ab are rarely reported (16, 17), but there were no such cases in our study. The preventive treatment profiles in our patients were consistent with previous international expert survey reports, with azathioprine and mycophenolate mofetil being the most commonly used drugs for maintenance (18). The MOG-Ab antibody titer may indicate severe relapses, but it is not necessarily indicative of long-term prognosis. Instead, it may be correlated with the time interval between the recent relapse and blood sampling. Negative seroconversion of MOG-Ab may indicate a lower activity of the disease (4, 19, 20).

In terms of the epitope of MOG-Ab, the P42 position in the MOG protein is known to be a major target of human MOG-Ab (21, 22). However, the clinical diversity associated with different MOG-Ab epitopes has not been extensively studied. One large study found that adults with relapsing ON were less reactive to the P42 position, while most MOG-Ab in pediatric patients recognized the P42 position. Our study also found that the P42 position in the MOG protein was the most frequently targeted by MOG-Ab, and patients with a monophasic clinical course and childhood-onset patients were only present in the group that showed reactivity to the P42 position (21). These findings are in line with previous reports; other clinical characteristics, including the total number of attacks, clinical presentation at onset, and phenotype of MOGAD, were not different according to the presence of reactivity to the P42 site. However, in our study, 41.7% of the samples showed no decreased reactivity to human MOG variants, which is a higher proportion than in previous reports conducted in Western countries (6). This may be due to the low affinity of MOG-Ab to certain epitopes, and the possibility of human leukocyte antigen (HLA) variation across different ethnic groups; there was no strong association with MOG-Ab in UK population, but the study conducted in the Chinese pediatric-onset cohort suggested the association between DQB1*05:02–DRB1*16:02 haplotype and MOGAD (23, 24).

The identification of epitopes in autoimmune disorders could be important. Knowledge of the autoantibody epitope target could lead to better diagnosis with precise testing and the development of epitope-specific medicines (25). Further large-scale studies that include clinical analysis of MOG-Ab and their epitopes are necessary to fully understand the clinical significance of MOG-Ab epitopes in patients with MOGAD.

Our study has several limitations that should be considered when interpreting our findings. First, the demographic profiles of enrolled patients were not controlled, as patients were recruited from a neurology clinic, which may have influenced the clinical characteristics of the MOGAD in this study and limited the number of childhood-onset patients. This limits the generalizability of our results. Second, a limited number of samples were collected due to patient consent, which may have restricted the ability to draw concrete conclusions about the relationship between the epitope and clinical features. Despite these limitations, this is the first study to examine the clinical significance of MOG-Ab epitopes in Korean patients, which is a strength of this study. However, further large-scale studies of MOG-Ab epitopes in Korean patients are needed. Third, this study is a cross-sectional analysis of MOG-Ab epitopes, and we did not evaluate the temporal stability of the epitopes or the change in the titer of MOG-IgG. Although a previous study showed that reactivity to P42 remained unchanged after 9 years of follow-up, temporal changes in the titers could be a crucial factor for predicting the clinical course of the disease (21, 26). Fourth, this study did not evaluate the radiological phenotypes and their correlation with P42 binding reactivity, although radiological features are one of the crucial clinical aspects of patients with MOGAD. Additionally, two of the patients included in the analysis met the diagnostic criteria of MS and were receiving DMT treatment at the time of sampling. Although MOG antibodies can be found in patients who have been diagnosed with MS, the antibodies tend to be more prevalent in children than in adults and can wane over time (27). Given the lack of subsequent antibody monitoring, our results should be approached with caution. Lastly, this study only evaluated MOG-Ab epitopes using serum samples, but recent studies have reported that some patients with MOGAD are positive for MOG-Ab only in their cerebrospinal fluid (CSF) samples, and an intra-individual heterogeneity of epitopes targeted by MOG-Ab from MOG-specific B cells was reported (28, 29). This suggests that there may be different sources of the production of MOG-Ab. Therefore, evaluations of MOG-Ab epitopes using both serum and CSF samples in patients with MOGAD may be necessary.

In conclusion, we developed an in-house cell-based immunoassay to analyze the epitopes of MOG-Ab in Korean patients with MOGAD. The results showed that the P42 site on the MOG protein is the primary target of MOG-Ab in Korean patients, and may have a correlation with the childhood-onset and monophasic clinical course of MOGAD. Further studies are needed to determine the predictive value of MOG-Ab and its epitopes.

## Data availability statement

The raw data supporting the conclusions of this article will be made available by the authors, without undue reservation.

## Ethics statement

The studies involving human participants were reviewed and approved by the institutional review board of the Samsung Medical Center. The patients/participants provided their written informed consent to participate in this study.

## Author contributions

JS: conceptualization, data investigation and analysis, visualization, and manuscript writing and editing. MJ: data curation and methodology. YC, HJ, HL, SK, and J-HM: data collection. E-SK: supervision and methodology. BK: conceptualization, data collection, data analysis, supervision, and manuscript review and editing. All authors contributed to the article and approved the submitted version.

## Funding

This research was supported by a grant from the National Research Foundation of Korea (NRF) funded by the Korean government (NRF-2019R1F1A1062978) and Soonchunhyang University Research Fund.

## Conflict of interest

J-HM is funded by and has received research support from the National Research Foundation of Korea and SMC Research and Development Grant. She has lectured, consulted, and received honoraria from Bayer Schering Pharma, Merk, Biogen Idec, Sanofi, UCB, Samsung Bioepis, Mitsubishi Tanabe, Celltrion, Roche, and Janssen. BK has received honoraria and/or consulting fees from Bayer, Genzyme, Merk, Celltrion, Astellas, Genuv, and Corestem.

The remaining authors declare that the research was conducted in the absence of any commercial or financial relationships that could be construed as a potential conflict of interest.

## Publisher’s note

All claims expressed in this article are solely those of the authors and do not necessarily represent those of their affiliated organizations, or those of the publisher, the editors and the reviewers. Any product that may be evaluated in this article, or claim that may be made by its manufacturer, is not guaranteed or endorsed by the publisher.
