# Supplementary material for: Clinical characteristics of myelin oligodendrocyte glycoprotein antibody-associated disease according to their epitopes
Source: Front Neurol. 2023 Jun 26;14:1200961. doi: 10.3389/fneur.2023.1200961 (PMC10331291; doi:10.3389/fneur.2023.1200961)
Supplement: Supplementary file 1 [file Data_Sheet_1.DOCX]

**Supplementary methods**

*In-house cell-based assay (CBA) for MOG-Ab detection*

For the cell-based immunoassay, HEK 293 cells were used, which were transfected overnight with a full-length human MOG using Lipofectamine 3000 reagent (Life Technologies, Thermo Fischer Scientific, Waltham, MA, USA) according to the manufacturer’s specifications. The transfected cells were washed with Dulbecco’s Modified Eagle Medium (DMEM; Thermo Fischer Scientific, Waltham, MA, USA) and incubated with serum samples diluted with 1% BSA in DMEM (1:20 dilution) for 60 min at room temperature, fixed with 4% paraformaldehyde for 1 min, washed three times with buffer, and then incubated with goat anti-human IgG1-specific antibody conjugated with AlexaFluor488 (Thermo Fischer Scientific, Waltham, MA, USA). After antibody labeling, cells were washed three times in phosphate-buffered saline (PBS) then evaluated under a fluorescence microscope (Eclipse 80i; Nikon; Tokyo Japan). Full-length human MOG protein expression in HEK 293 cells was confirmed by western blotting with a commercial anti-MOG antibody (Santa Cruz Technology, Dallas, TX, USA). When scoring MOG-Ab seropositivity, the investigator was blinded to the clinical and laboratory information of the patients. The descriptions of the intensity of surface immunofluorescence are: 0, There is no binding around the cell membrane, and the staining does not form a ring around the cell membrane; 0.5, There is a weak binding around the cell membrane but not surrounding the entire membrane; 1, There is clear but weak binding that surrounds the entire membrane; 2, The entire membrane is clear and moderately bright; 3, The binding is clear and brilliant, like the brightness of the positive control; 4, Very bright binding stronger than the positive control. The presence of MOG-Ab was confirmed if the intensity was 1+ or stronger.

*Generation of human MOG variants and molecular cloning*

Seven human MOG variants were synthesized by a commercial gene synthesis service (Bioneer, Daejeon, Korea): R9G/H10Y, N31D, P42S, R86Q, H103A/S104E. The oligonucleotides used were:

5’–CAGAGTGATAGGACCAGGATACCCTATCCGGGCTCTGG–3’ (R9G/H10Y),

5’–CATATCTCCTGG­GAAGGACGCTACAGGCATGGAGG–3’ (N31D),

5’–GTGGGGTGGTACAGATCTCCCTTCTCTAGG–3’ (P42S),

5’–GGTGACTCTCAGGATCCAGAATGTAAGGTTCTCAGATG–3’ (R86Q),

5’–CCTGCTTCTTCC-GAGATGCTGAATACCAAGAGGAGGCAG–3’ (H103A/S104E).

Each gene was cloned into the pIRES2 DsRed-Express2 vector (Takara, Shiga, Japan) by cloning (Bioneer, Daejeon, Korea). Polymerase chain reaction (PCR) was performed using the AccuRapidTM Cloning kit (Bioneer, Daejeon, Korea) inserted between the *EcoRI* and *PstI* sites of the plasmid. All sequences of the purified plasmids were analyzed by DNA sequencing (Bioneer, Daejeon, Korea). The resulting plasmids were used for the transformation of the DH5a competent cells (Thermofisher, Waltham, MA, USA). Then, the *E. coli* was cultured. Plasmid DNA was extracted from cultured cells using a plasmid DNA purification kit (Qiagen, Germantown, MD, USA). Plasmid DNA was quantified using a NanoDrop spectrophotometer (Thermo Scientific, Waltham, MA, USA).

*Western blot and FACS analysis*

The hMOG or each of the hMOG variants was transfected with Lipofectamine 3000 (Life Technologies, Carlsbad, CA, USA) to HEK293 cells overnight. Transfected HEK293 cells were harvested and lysed with Ripa buffer (Thermo Fisher, Waltham, MA, USA). The extracted total protein was separated by electrophoresis on a NuPAGE Bis-Tris gel system. The separated protein gel was transferred to a PVDF membrane with an iBlot system (Thermo Fisher, Waltham, MA, USA). The protein transferred membrane was incubated overnight at 4 °C with an anti-MOG antibody (Santa Cruz Biotechnology, USA) at 1:2,000 dilutions. The blots were washed and incubated with an anti-mouse HRP-conjugated secondary antibody (Santa Cruz Biotechnology, USA) at 1: 5,000 for 2 h at room temperature. The washed membrane was developed with ECL (Santa Cruz Biotechnology, Dallas, TX, USA).

Transfected cells were incubated with each patient’s serum at a dilution of 1:20 for 1 h at room temperature and then developed with Alexa Flour 488 conjugated anti-human IgG1 (Thermo Fisher, Waltham, MA, USA) for imaging with a fluorescence microscope (Eclipse 80i, Nikon, Tokyo, Japan) or mouse anti-human IgG1 (Thermo Fisher, Waltham, MA, USA) with Alexa Fluor 647 conjugated anti-mouse antibody (Thermo Fisher, Waltham, MA, USA) at 1:500 for flow cytometry (Verse, BD, Franklin Lakes, NJ, USA). Flow cytometry data were analyzed using the FACS Suite (BD, Franklin Lakes, NJ, USA).

**Supplementary Figure**

Supplementary Figure 1. Confirmation of the expression of human MOG and variants

**
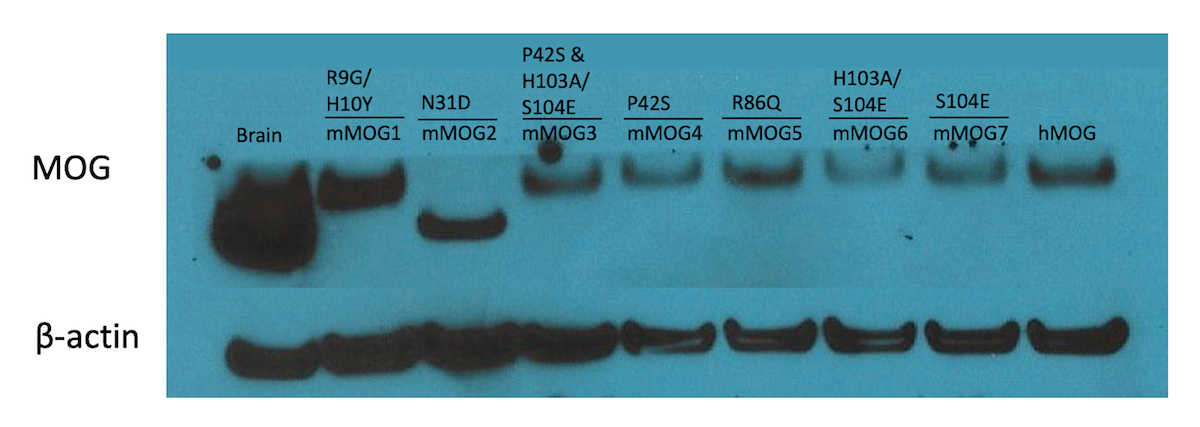
**Western blotting showed human MOG and human MOG variants expression in the transfected HEK293 cell line.

MOG, myelin oligodendrocyte glycoprotein; mMOG, mutant MOG; hMOG, human MOG
